# High Genetic Diversity Among *Bacillus cereus* Isolates Contaminating Donated Milk at a Canadian Human Milk Bank

**DOI:** 10.3390/microorganisms13051136

**Published:** 2025-05-15

**Authors:** Mathew Gene, Jennifer L. Guthrie, Kevin Li, Sarah Teatero, Aimee Paterson, Angel Li, Alain Doyen, Deborah Yamamura, Sarah Khan, Jocelyn A. Srigley, Debbie Stone, Deborah L. O’Connor, Susan Poutanen, Sharon Unger, Allison McGeer, Nahuel Fittipaldi

**Affiliations:** 1 Department of Cell and Systems Biology, University of Toronto, Toronto, ON M5S 3G5, Canada; mathew.gene@alumni.utoronto.ca; 2 Public Health Ontario, Toronto, ON M5G 1M1, Canada; jennifer.guthrie@uwo.ca (J.L.G.); sarah.teatero@oahpp.ca (S.T.); 3 Department of Microbiology and Immunology, Western University, London, ON N6A 3K7, Canada; 4 GREMIP and CRIPA, Faculty of Veterinary Medicine, University of Montreal, St-Hyacinthe, QC J2S 2M2, Canada; kevin.li.2@umontreal.ca; 5 Sinai Health System, Toronto, ON M5G 1X5, Canada; aimee.paterson@auckland.ac.nz (A.P.); xinliu.angelli@sinaihealth.ca (A.L.); debbie.stone@sinaihealth.ca (D.S.); susan.poutanen@sinaihealth.ca (S.P.); allison.mcgeer@sinaihealth.ca (A.M.); 6 Department of Food Sciences, Institute of Nutrition and Functional Foods (INAF) and Dairy Science and Technology Research Center (STELA), Laval University, Quebec City, QC G1V 0A6, Canada; alain.doyen@fsaa.ulaval.ca; 7 Microbiology Department, Hamilton General Hospital, Hamilton, ON L8L 2X2, Canada; yamamura@hhsc.ca; 8 Department of Pathology and Molecular Medicine, Faculty of Health Sciences, McMaster University, Hamilton, ON L8S 4L8, Canada; 9 Division of Pediatric Infectious Diseases, McMaster Children’s Hospital, Hamilton Health Sciences, Hamilton, ON L8N 3Z5, Canada; khan259@mcmaster.ca; 10 Department of Pediatrics, Faculty of Health Sciences, McMaster University, Hamilton, ON L8S 4L8, Canada; 11 Department of Pathology & Laboratory Medicine, BC Children’s Hospital & BC Women’s Hospital + Health Centre, Vancouver, BC V6H 3N1, Canada; jocelyn.srigley@cw.bc.ca; 12 Department of Pathology & Laboratory Medicine, Faculty of Medicine, University of British Columbia, Vancouver, BC V6T 1Z3, Canada; 13 BC Children’s Hospital Research Institute, Vancouver, BC V5Z 4H4, Canada; 14 The Rogers Hixon Ontario Human Milk Bank, Toronto, ON M5G 1X5, Canada; sharon.unger@iwk.nshealth.ca; 15 Department of Nutritional Sciences, University of Toronto, Toronto, ON M5G 1A8, Canada; deborah.oconnor@utoronto.ca; 16 Department of Laboratory Medicine and Pathobiology, University of Toronto, Toronto, ON M5S 3K3, Canada; 17 IWK Health Centre, Halifax, NS B3K 6R8, Canada; 18 Department of Paediatrics, University of Toronto, Toronto, ON M5G 1X8, Canada

**Keywords:** *Bacillus cereus*, human milk bank and hospital, human milk, holder pasteurization, contamination routes, genetic diversity, whole-genome sequencing, sporulation, neonatal health

## Abstract

*Bacillus cereus* poses a persistent challenge for human milk banks (HMBs) due to its ability to survive Holder pasteurization (HoP; 62.5 °C for 30 min). To ensure neonatal safety, any milk found to be contaminated post-HoP must be discarded, which impacts milk supply and adds to the operational demands of HMBs. In this study, we analyzed 688 *B. cereus* isolates from human milk (pre- and post-HoP), as well as from patient and environmental sources, to investigate human milk contamination by *B. cereus* at a Canadian HMB. Despite the limited temporal and geographic scope of the collection, the isolates exhibited remarkable genomic diversity, comparable to global *B. cereus* collections. Phylogenetic analysis at the core genome level revealed no clear clustering by isolate source, suggesting multifactorial pathways of *B. cereus* contamination. Isolates surviving HoP displayed gene variants linked to sporulation and cell wall integrity, suggesting a potential basis for HoP tolerance. Our findings emphasize that while genomic analyses offer major valuable insights, they alone are insufficient to address the complexities of *B. cereus* contamination in HMBs. Addressing this challenge will require combining genomic tools with robust monitoring systems, improved human milk-handling protocols, and pasteurization strategies better-suited to countering *B. cereus* resilience.

## 1. Introduction

Preterm birth accounts for approximately 8% of the 350,000 children born annually in Canada [1]. These vulnerable patients often require prolonged hospitalization and are at increased risk of complications, making breastmilk an essential health-promoter due to its nutritional, immunological, and developmental benefits [2,3]. However, preterm babies may be hospitalized apart from their mothers, or mothers may not be able to produce an adequate milk supply if they are ill themselves, under stress, and/or have an immaturity of the mammary secretory gland due to preterm delivery [3]. Human milk banks (HMBs) help to mitigate these issues by collecting breastmilk from lactating donor women, pasteurizing it, and distributing it via prescription to preterm babies in neonatal intensive care units (NICUs).

Most HMBs process donor milk using Holder pasteurization (HoP), which involves heating the milk to 62.5 °C for 30 min to reduce the bacterial load. While this process is effective against most bacterial contaminants, one fundamental challenge faced by HMBs is contamination with *Bacillus cereus* sensu stricto (for simplicity, henceforth referred to as *B. cereus*), a ubiquitous environmental contaminant but also a potential cause of devastating neonatal invasive infections [4,5]. This organism can survive HoP due to its ability to form endospores [6]. If these spores germinate post-pasteurization, they can potentially multiply and pose a safety risk to neonates. Human milk from three to five or more individual donors is typically pooled into batches and blended to ensure constituent variation in the milk. If *B. cereus* is present in any donation, this can lead to its presence post-HoP and can force the disposal of entire batches of donor milk, jeopardizing supply. This practice significantly impacts HMBs, which globally lose 15 to 30% of human milk donations due to *B. cereus* contamination [3].

Contamination by *B. cereus* may stem from multiple sources, including colonized donors, environmental exposure, or human milk-handling procedures. In addition to sporulation, the ability of *B. cereus* to form biofilms enhances its persistence in the environment and contributes to its resistance to standard cleaning and disinfection protocols [7]. Current detection techniques, such as bacterial culturing on selective media followed by biochemical identification, cannot distinguish between different sources of contamination [4,8,9]. Moreover, common molecular tests are not useful in most cases, as this bacterium is phylogenetically indistinguishable from other phenotypically distinct species within the *Bacillus cereus* sensu lato group [6,8,9,10]. Additionally, little is known about the factors contributing to *B. cereus*’ resistance to pasteurization or whether strains from different sources exhibit similar resistance capacities. Whole-genome sequence (WGS)-based approaches offer a powerful way to investigate these questions by characterizing the genetic relationships between *B. cereus* strains and identifying genomic features associated with their survival and persistence post-pasteurization.

Here, we hypothesized that the discriminatory power of WGS could be leveraged to differentiate *B. cereus* strains contaminating human milk from those found in other sources, such as human patients or the environment. Specifically, we aimed to characterize the genomic relationships between *B. cereus* strains from different sources to establish a baseline regarding their genetic diversity and population structure and to identify candidate genes associated with persistence in human milk. Our results provide data-driven insights that could enhance contamination-mitigation strategies in HMBs.

## 2. Materials and Methods

### 2.1. Sampling and Culturing-Dependent Screening

We used a collection of 744 isolates recovered between February 2017 and May 2018, identified as belonging to the *B. cereus* group using Matrix-Assisted Laser Desorption/Ionization-Time of Flight (MALDI-TOF) mass spectrometry (MS)-based VITEK-MS (bioMérieux, Marcy-l’Étoile, France), operated with the commercially available Knowledge Base V3.2. The isolates were obtained from various sources, and we included multiple isolates per plate when colonies were observed with diverse visible phenotypes, such as those with variations in their color, size, surface texture, or hemolytic capacity, or those with colony-spreading suggestive of swarming motility.

Of these 744 isolates, 217 were recovered from donated human milk, including 24 isolates from 15 plates collected before HoP and 193 isolates from 83 plates collected after HoP at the Rogers Hixon Ontario HMB, which operates within the premises of Mount Sinai Hospital (MSH) in Toronto, ON, Canada. Metadata detailing the date of isolation, colony phenotypes, and source were recorded for pre- and post-pasteurization milk isolates as part of routine HMB intake protocols. Similar metadata were also collected for environmental isolates, defined as *B. cereus* strains recovered from capture plates placed in the HMB premises, the hospital NICU, other hospital wards, and the MSH Microbiology Laboratory, as well as from swabs of preparation benches and equipment at the HMB, personal protective gowns used by HMB personnel, and unused breast pads provided by some milk donors. This group included 131 isolates recovered from 23 plates. Patient isolates included strains obtained from routine nasal, rectal, axilla, groin, and perineum diagnostic swabs collected from individuals receiving care at three acute care hospitals in downtown Toronto, all served by the MSH Microbiology Laboratory. These isolates are referred to hereafter as the “colonization” isolates and comprised 247 isolates from 52 plates. Additionally, we included isolates recovered from blood, bone, lung, and autopsy tissue samples from individuals admitted to the same three hospitals with clinical signs compatible with bacterial infection. These are referred to hereafter as the “clinical” isolates and comprise 149 isolates from 53 plates. In line with standard clinical microbiology protocols, multiple colonies were often subcultured from the same diagnostic plate, even when they appeared to be morphologically similar, to rule out mixed flora or contamination. This approach was preserved in our study to capture potential within-sample variation. Case-level clinical metadata for these *B. cereus* isolates were obtained from routine diagnostic procedures and corresponding chart reviews. For visualization and comparative analyses in figures and tables, colonization and clinical isolates were grouped under the category “Patient”, without distinction between colonization or infection status.

### 2.2. DNA Extraction and Whole-Genome Sequencing

For DNA preparation, pure cultures of isolates were grown overnight on Columbia agar plates containing 5% sheep blood at 37 °C with 5% CO_2_. Bacteria were harvested by washing the plates with 1 mL of TrisEDTA buffer, pH 8. A total of 600 μL of bacterial suspension was then added to tubes containing 0.1 mm silica beads (Bertin Technologies SAS, Montigny-le-Bretonneux, France) and lysed at 4500 rpm for 10 s using a Precellys 24 tissue homogenizer (Bertin Technologies SAS). Lysates were centrifuged for 2 min at 14,000× *g* and DNA was prepared from resulting supernatants by using the Qiagen DNA mini kit (Qiagen, Toronto, ON, Canada), following the manufacturers’ instructions for Gram-positive organisms. Genomic libraries were prepared using Nextera XT Library Prep Kits (Illumina, San Diego, CA, USA) according to the manufacturer’s protocol, and sequenced as paired-end (150 + 150 bp) reads on the Illumina HiSeq4000 platform at Génome Québec Innovation Centre sequencing facilities (Montreal, QC, Canada).

### 2.3. Bioinformatics Analysis and Statistical Analysis

Onboard Illumina software (HiSeq Software Suite v.3.3) was used to determine quality scores, parse the multiplexed sequencing reads, and remove barcode information. The A5 pipeline V.20160825 [11] was used to generate de novo assemblies. Genomes with initial Q scores < 30, and those failing to assemble in fewer than 500 contigs were discarded. Assembled contigs were annotated with Prokka v.1.12 [12]. Species identification was assessed with Kraken v.2 [13]. Pangenome and core genome analysis was performed using Roary [14], version 3.13.0, with a minimum BLASTP v.2.6.0 identity of 95%. Genes present in ≥99% of genomes were defined as core. Accessory genes were categorized based on presence frequency as follows: soft core (≥95% but <99%), shell (≥15% but <95%), and cloud (<15%). Gaps in the core genome alignment sequences were removed with trimAl v.1.2rev59 [15], while regions of high recombination were identified and removed with Gubbins [16]. To calculate a pairwise single-nucleotide polymorphism (SNP) matrix in each of the genomes, we used snp-dist v.0.6 [17]. Maximum-likelihood phylogenetic trees were built with FastTree 2.1 using the generalized time-reversible model along with the gamma-distributed site-rate heterogeneity over 1000 bootstrap replications [18]. A reduced subset of assembled *B. cereus* genomes (n = 257) was used to minimize potential noise from multiple genomes originating from the same plate (see results). Phylogenetic trees were visualized with the ggtree package v.2.1 in R [19]. Clustering based on the core genome SNPs was performed through a hierarchical Bayesian analysis of population structure (BAPS), as implemented in the R package rhierBAPS v.1.1 [20]. Genome-wide association studies (GWAS) were performed using pyseer v.1.3.5 [21] on the reduced subset of 257 *B. cereus* genomes. Within pyseer, a unitig-based association analysis was conducted using a mixed-effects model. Unitigs, derived from k-mers and counted with a unitig-counter, were used to reduce sequence redundancy and mitigate confounding effects [22]. Population structure effects were corrected using a recombination-removed core genome approximately maximum-likelihood phylogenetic tree calculated using FastTree 2.1 as input to pyseer. Unitigs were filtered using a significance threshold of 1.90 × 10^−9^ and mapped to the reference genome of *B. cereus* ATCC 14579 (GenBank accession number NZ_CP138336.1), as well as to additional representative assemblies, to annotate the significant unitigs, which were visualized using ggplot2 in R (3.6.3) [23]. All statistical analyses were conducted in R (version 3.6.3). The Wilcoxon test was used to compare within- and between-plate median pairwise SNP distances.

## 3. Results

### 3.1. Species Typing of B. cereus Group Isolates

We sequenced the genomes of 744 isolates deemed to belong to the *B. cereus* group by MALDI-TOF MS. Of these, 49 did not meet our quality check thresholds and were excluded from further analysis. This included 12 genomes whose Q scores were <30 and 37 genomes that either failed to assemble de novo or whose de novo assemblies exceeded our threshold of <500 contigs, likely due to challenges such as highly repetitive genomic regions or uneven sequencing coverage (Figure S1). Although the excluded genomes represented 6.59% of the dataset, they were removed based solely on predefined quality criteria, without enrichment for any particular isolate source (e.g., milk, patient, or environmental), minimizing the risk of introducing bias and ensuring the reliability of the downstream analyses and conclusions.

We used Kraken to confirm the results of the MALDI-TOF MS speciation of the remaining 695 isolates. For seven isolates, MALDI-TOF and Kraken were not in agreement, with Kraken assigning the discordant isolates to *Myroides odoratus* (n = 5), *Aeromonas veranii* (n = 1), and *Micrococcus luteus* (n = 1). The discrepancy between MALDI-TOF MS and Kraken likely reflects methodological differences in taxonomic resolution and reference database structure. MALDI-TOF assigns species based on spectral patterns of abundant proteins and is constrained by the breadth of its spectral library, whereas Kraken uses k-mer-based whole-genome comparisons, offering higher resolution but potentially introducing bias due to uneven database representation. For example, of the 688 remaining isolates, Kraken identified 16 as being *Bacillus thuringiensis*, a major biopesticide control agent that produces plasmid-encoded bioinsecticidal crystal proteins [10,24], and 6 as *Bacillus wiedmanii*, an organism frequently isolated from dairy products and/or dairy environments [25]. At the chromosomal level, the differences between *B. cereus* and *B. thuringiensis* are minimal and clusters formed by the type strains of *B. cereus* sensu stricto and *B. thuringiensis* have been documented [26,27]. Since we did not detect the genes encoding the crystal toxins characteristic of *B. thuringiensis* in the genomes of the 16 isolates identified by Kraken as *B. thuringiensis*, we retained these genomes for further downstream analyses. We also retained the genomes of *B. wiedmanii* isolates to ensure a comprehensive representation of organisms frequently associated with dairy environments in our dataset [25]. Thus, our final collection comprised a total of 688 genomes obtained from human milk and the hospital environment at MSH, along with colonization and clinical isolates collected from patients receiving care at three acute care hospitals in Toronto, all served by the MSH laboratory. The average sequencing depth of coverage was 91.55× (range of 49.89×–217.64×). Table S1 summarizes the origin and time of collection of these isolates, while Table S2 provides NCBI Sequence Read Archive accession numbers for the 688 genomes.

### 3.2. Core and Pangenome of the B. cereus Collection

To begin to characterize the *B. cereus* population structure, we built the pangenome of our isolate collection using Roary [14]. We identified 63,199 protein-coding gene sequence (CDS) clusters, representing groups of homologous CDSs, across the 688 genomes, with, on average, 5840 CDSs per input genome. The shortest CDS cluster sequence was 69 nt, the longest was 19,196 nt, and the average length of a CDS cluster was 719 nt. The average difference between the shortest and longest CDS in a cluster was only 146 nt, indicating that the input data were relatively uniform and consistent with inputs derived from complete genomes. Henceforth, for simplicity, we refer to Roary-identified CDS clusters as ‘genes’. We defined the core genome as the genes present in at least 99% of isolates. Using this criterion, the core genome of the isolate collection was composed of 1933 genes (~3% of all genes). Accessory genes were divided into 49,727 shared accessory genes (i.e., non-core genes present in at least two isolates; ~70.9% of all genes) and 14,479 unique accessory genes (i.e., genes present in only one isolate; ~29.1% of all genes). Table S3 summarizes the distribution of genes across different presence thresholds in the *B. cereus* pangenome.

As additional genomes were incorporated into the analysis, new genes continued to accumulate at a steady rate, with no evidence of saturation in the total gene pool. To formally assess whether the pan-genome of this *B. cereus* collection was open or closed, we applied a power-law regression model of the form Ps = κn^γ^ where Ps is the total number of genes (pangenome size), n is the number of genomes analyzed, κ is a fitted constant, and γ is the exponent that characterizes pangenome openness. The resulting value of γ ≈ 0.447, estimated from our gene accumulation data, supports the conclusion that the *B. cereus* pangenome remains open. The fitted curve is shown in Figure S2. This result highlights the substantial genetic diversity present within a geographically and temporally restricted collection of isolates, sampled from a single human milk bank and nearby healthcare facilities. Given these findings, we next aimed to rapidly assess the phylogenetic relationships among the 688 isolates. To achieve this, we constructed a phylogenetic tree based on the binary presence and absence of genes across the pangenome, as determined using Roary’s gene presence/absence matrix. While we did not exclude mobile genetic elements (MGEs) or recombination-prone regions, which are subject to horizontal transfer (HGT) and can potentially confound phylogenetic inference, this approach has been shown to effectively reproduce the basic phylogenetic structure of *B. cereus* [28].

**Figure 1:**
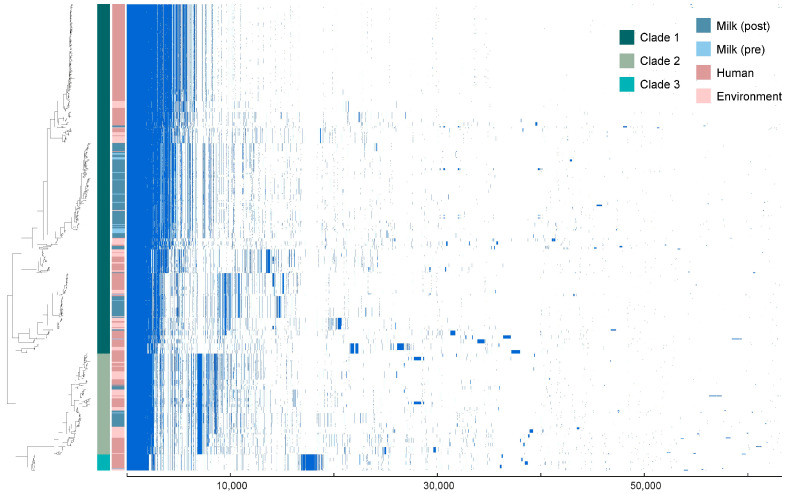
Phylogenetic relationships and pangenome of 688 *B. cereus* strains used in this study. The phylogenetic tree, on the left of the Figure, was constructed using the gene presence/absence matrix generated by Roary for the 63,199 genes in the pangenome of the collection. Overall, the three main clades associated with *B. cereus* [10] were recapitulated, with human milk-derived isolates distributed across all three clades. The pangenome map displays the presence/absence of the 63,199 genes among the isolates, arranged from left to right, starting with core genes, followed by shared accessory genes, and ending with unique genes.

Although this preliminary analysis recapitulated the three major previously described *B. cereus* clades [10], a key observation was the lack of clustering of isolates by collection source. While a substantial proportion of milk isolates appeared in one branch of Clade 1, milk (pre- and post-pasteurization), human patients, and environmental sources were intermingled across all phylogenetic clades, revealing no clear origin-specific patterns (Figure 1). This lack of segregation underscores the complex and interconnected nature of the *B. cereus* population, even within a relatively confined spatial and temporal framework.

### 3.3. Analysis of Core Genome SNP Distances Between Isolates

While the accessory gene-based phylogenetic analysis provided valuable insights into the population structure of the isolates, its potential variability introduced through horizontally acquired regions and the absence of certain genes in some isolates. These factors could obscure the true evolutionary relationships. To address these limitations, we performed a core genome SNP analysis aimed at improving the assessment of the genetic diversity and relatedness of the isolates.

**Figure 2:**
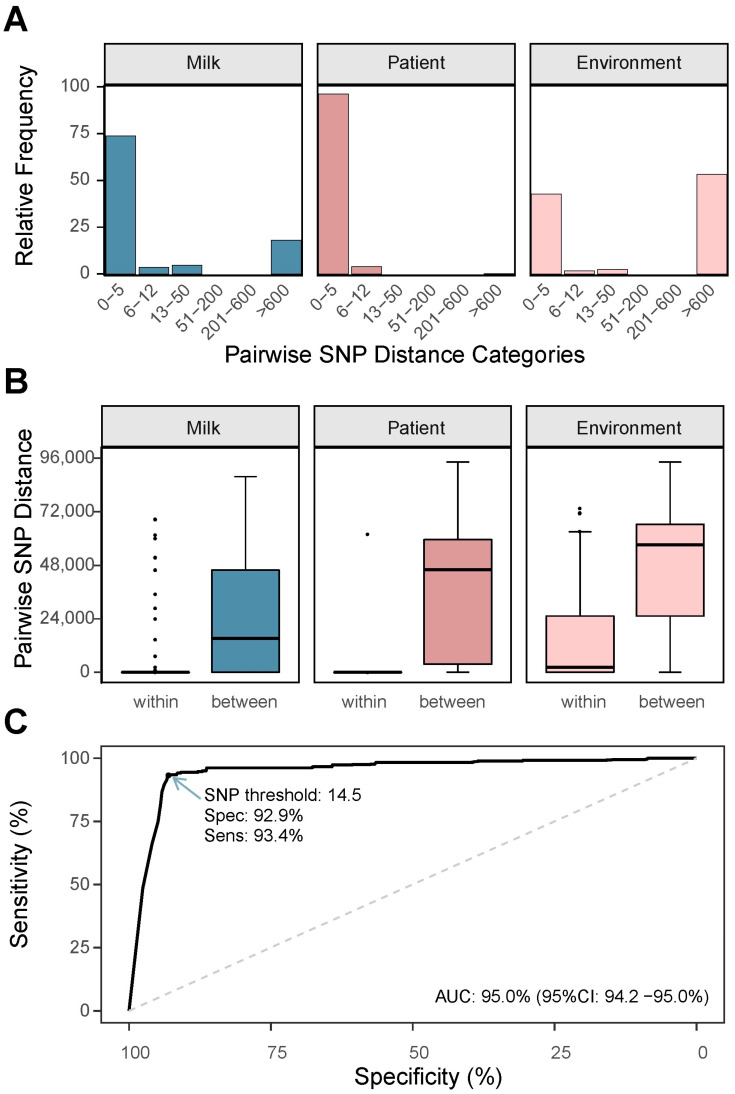
Analysis of pairwise SNP distances and classification of near-identical isolates. (**A**) Relative frequency distribution of pairwise SNP distances within the same plate, categorized by specimen type (human milk, patient, and environment). Human milk and environmental isolates displayed a bimodal distribution (0–50 SNPs and >600 SNPs), while patient isolates showed a unimodal distribution with distances primarily between 0 and 12 SNPs. The narrow distribution among patient isolates reflects our sampling strategy, which involved sequencing multiple colonies from the same diagnostic plate. (**B**) Boxplots comparing pairwise SNP distances within the same plate (“within”) and across different plates (“between”) for human milk, patient, and environmental isolates. Isolates from the same plate displayed significantly lower distances compared to those from different plates (*p* ≤ 0.05). (**C**) A receiver operating characteristic (ROC) curve was used to identify a 14.5-SNP threshold for classifying near-identical isolates on the same plate. The SNP threshold had a sensitivity of 93.4% and a specificity of 92.9%, with an area under the curve (AUC) of 95.0% (95% CI: 94.2–95.0%).

We began by analyzing pairwise SNP distances according to isolate source. Human milk- and environmental-derived isolates displayed a bimodal distribution of SNP distances (0–50 and >600 SNPs), indicating the presence of both closely and distantly related isolates within these groups (Figure 2A). In contrast, patient-derived isolates showed a much narrower range of SNP distances, with the majority of comparisons falling within 0 to 12 SNPs. This pattern primarily reflects our sampling strategy rather than the underlying population structure. For patient samples, multiple colonies were subcultured from the same diagnostic plate during routine laboratory procedures, even when colonies appeared morphologically similar. This approach, standard practice in clinical microbiology to rule out mixed flora or contamination, resulted in the inclusion of genetically near-identical isolates from the same patient sample. Similarly, for milk and environmental samples, multiple colonies were selected per plate; however, in these cases, colonies were deliberately chosen based on distinct visible phenotypes, leading to a higher likelihood of genetic unrelatedness among isolates from the same plate.

We further compared pairwise SNP distances between isolates originating from the same plate and those obtained from different plates. As expected, same-plate comparisons showed significantly lower SNP distances (*p* ≤ 0.05), regardless of isolate source (Figure 2B). Among patient isolates, same-plate pairs had a mean SNP distance of 1.3, reflecting within-sample homogeneity. In contrast, milk and environmental isolates from the same plate showed much higher mean SNP distances (4105 and 10,841 SNPs, respectively), consistent with our strategy of selecting phenotypically diverse colonies likely representing unrelated strains (Table S4).

Recognizing that our sampling approach introduced redundancy in the dataset, particularly among patient isolates, we implemented an iterative filtering strategy to reduce the overrepresentation of near-identical genomes while preserving meaningful genetic diversity. To distinguish near-identical isolates from distantly related isolates, we performed a receiver operating characteristic (ROC) curve analysis to define the SNP distance threshold. This analysis identified a cutoff of 14.5 SNPs for classifying isolates as near-identical (Figure 2C).

We applied this threshold to select representative genomes from multi-isolate plates. For example, on a plate with five isolates, there would be ten pairwise SNP distance comparisons. If all distances were below 14.5 SNPs, only one genome—typically the one with the highest-quality de novo assembly—was retained. If pairwise distances exceeded 14.5 SNPs, isolates were grouped accordingly, and one representative genome from each group was selected. For instance, if five isolates formed two groups, with one group containing two closely related isolates (<14.5 SNPs) and the other containing three (<14.5 SNPs), one genome from each group was retained.

Using this iterative approach, we retained one isolate per single-isolate plate and, for multi-isolate plates, one or more isolates depending on the observed genetic diversity. In the end, more than one isolate was retained from 17% of multi-isolate plates. This filtering process resulted in a non-redundant dataset comprising 257 genomes, which was used for all downstream analyses (Figure S1).

### 3.4. Accessory Gene Content Similarity and Its Relationship with Core Genome SNPs

The relationship between core genome diversity and accessory gene content provides important insights into the evolutionary and functional dynamics of bacterial populations. Since accessory genes are typically more variable and may be subject to horizontal gene transfer, we hypothesized that closely related isolates (as measured by core genome SNP distances) would share more similar accessory gene profiles. Conversely, distantly related isolates were expected to exhibit lower accessory gene content similarity. To test this hypothesis, we investigated whether there was an inverse relationship between pairwise core genome SNP distances and accessory gene content similarity.

**Figure 3:**
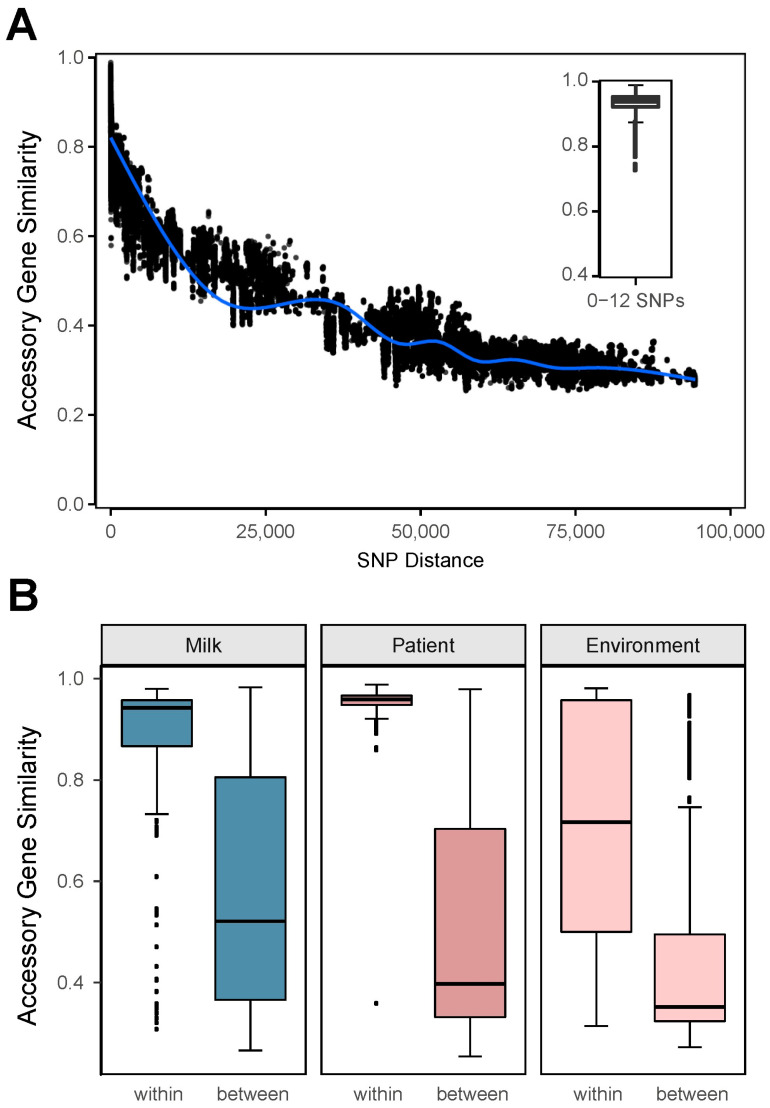
Relationship between core genome SNP distances and accessory gene content similarity. (**A**) Scatterplot showing the inverse relationship between pairwise core genome SNP distances and accessory gene content similarity across all isolate pairs. The blue line represents a smoothed trend line, highlighting the overall decline in accessory similarity with increasing SNP distance. The inset boxplot illustrates the high accessory gene content similarity (mean = 0.98) among isolate pairs with SNP distances in the 0–12 range. (**B**) Boxplots comparing accessory gene content similarity within the same plate (“within”) and across different plates (“between”) for human milk, patient, and environmental isolates. Isolates from the same plate showed significantly higher accessory similarity compared to those from different plates across all specimen types, with environmental isolates showing the largest variability (interquartile range (IQR) = 0.48).

An accessory similarity of 1 indicates that all accessory genes between a pair of isolates are identical, whereas a similarity of 0 means that no accessory genes are shared. Consistent with our hypothesis, we observed an inversely proportional relationship between core genome pairwise SNP distance and accessory gene content similarity (Figure 3A). Notably, within the 0–12 SNP range, accessory gene content similarity was 0.98, indicating that closely related isolates share highly similar accessory gene profiles. These results suggest that core genome SNP distances are a good predictor of accessory gene content, although the underlying genetic mechanisms driving this phenomenon remain to be elucidated.

We next examined accessory gene content similarity specifically within isolates from the same plate versus those from different plates to assess whether the trends observed in the core genome pairwise SNP analysis were reflected in the accessory gene content. As expected, accessory gene content similarity was higher among isolates from the same plate compared to isolates from different plates across all specimen types (Figure 3B). Among patient isolates, accessory gene content similarity had the lowest variation, with a standard deviation (SD) of 0.096, reflecting their genetic homogeneity. In contrast, human milk isolates exhibited an SD of 0.179, while environmental isolates showed the greatest variability, with an SD of 0.188. Environmental isolates also had the largest interquartile range (IQR = 0.48), indicating greater accessory gene diversity compared to human milk and patient isolates. This diversity likely reflects the broader variability and mixed phenotypic characteristics we observed on environmental plates.

### 3.5. Absence of Source-Specific Clustering Among B. cereus Isolates

**Figure 4:**
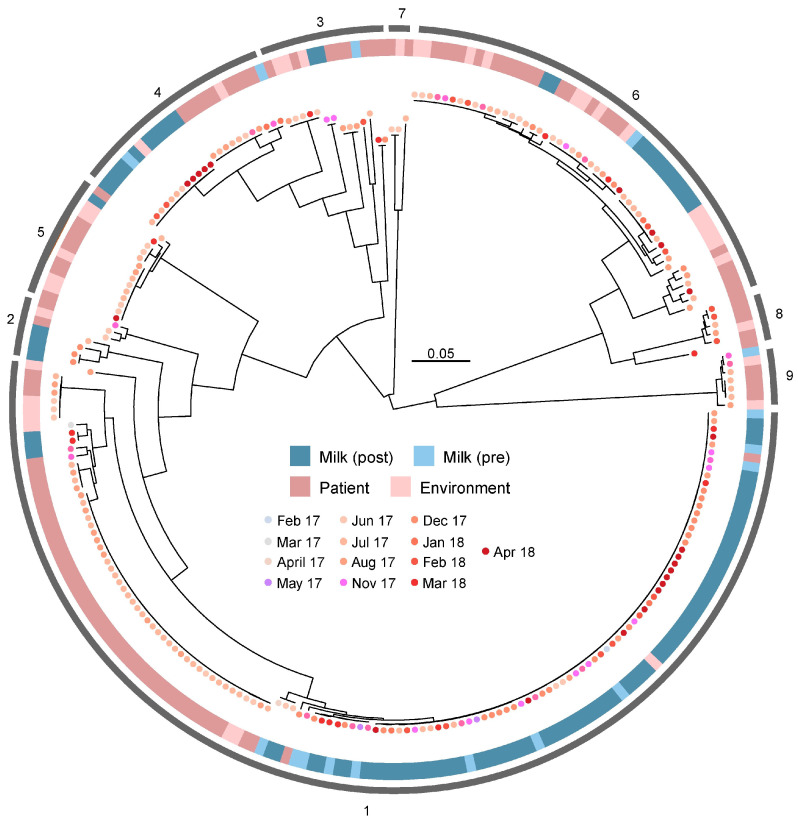
Phylogenetic relationships between 257 *B. cereus* isolates recovered from a human milk bank and three acute care hospitals in Toronto, Canada. The phylogenetic tree was constructed using a recombination- and gap-filtered core genome alignment of 309,814 nt derived from Roary. Branch lengths represent genetic distances based on SNPs, and the reliability of tree splits was estimated using the Shimodaira–Hasegawa test with 1000 resamples in FastTree 2.1. Hierarchical Bayesian clustering (BAPS) identified nine distinct genetic clusters, which are indicated in the outermost ring. The second outermost ring indicates the isolate source (human milk pre-pasteurization, human milk post-pasteurization, patient, and the environment), and the month of collection is represented by colored dots. Temporal data are distributed evenly across the tree, with no apparent overrepresentation of specific collection periods in any BAPS cluster. The scale bar indicates genetic distance based on core genome SNPs.

We next constructed a maximum likelihood phylogenetic tree for the reduced set of 257 genomes, using SNPs identified in the gap- and recombination-removed core genome alignment defined by Roary. This approach aimed to refine the understanding of genetic relationships by reducing the confounding effects potentially introduced by HGT events. Statistical support for phylogenetic clades was provided through Bayesian analysis, which clusters isolates based on genetic similarity. The phylogenetic tree revealed that human milk, patient, and environmental isolates were interspersed and were not exclusively associated with any specific BAPS cluster (Figure 4). This absence of clustering by isolation source further confirmed that *B. cereus* populations in the study setting are genetically highly diverse, regardless of their origin. However, of the 257 isolates, 134 (52.1%) clustered into BAPS cluster 1. Human milk-derived isolates were predominantly found in this cluster, with 70.2% (80 of 114) of human milk isolates assigned to cluster 1 (Figure 4).

The second-largest group, BAPS cluster 6, comprised 49 isolates (20.0% of the reduced dataset) and included 26.5% human milk, 44.0% patient, and 28.6% environmental isolates, reflecting a more balanced distribution of isolate sources (Figure 4). Clusters 7 and 8 were notable in that they did not contain any human milk-derived isolates, whereas patient isolates showed moderate clustering within specific groups. For instance, 43.4% (46 of 106) of patient isolates were concentrated in a single BAPS cluster (Figure 4). In contrast, environmental isolates were distributed across the phylogenetic tree, with at least one environmental isolate found in each of the nine BAPS clusters. Most environmental isolates (26 of 32, 81.3%) were located outside of cluster 1 (Figure 4).

To assess whether temporal biases in isolate collection influenced the observed population structure, we overlaid temporal data on the phylogenetic tree. Human milk-derived specimens were collected consistently over the 16-month study period, except for three months at the start of the collection and one month at the end (specifically for the pre-pasteurization subcategory, Table S1). In contrast, patient- and environmental-derived specimens had more sporadic collection patterns. Patient isolates were absent for five consecutive months at the beginning of the collection period, and within the non-clinical subcategory, there were an additional five months with no specimens collected (Table S1). For environmental isolates, the majority of specimens from each subcategory (e.g., breast pads, human milk bank equipment, NICU surfaces, lab areas, and hospital wards) were collected over a relatively short temporal span (Table S1). Despite these temporal limitations, the population structure appeared robust to the sparsity of certain isolate types. Hierarchical BAPS clusters were well distributed across the collection timeframe, with no specific months being overrepresented in any given cluster (Figure 4). Within BAPS cluster 1, all months of collection were represented, although 24.6% (33 of 134) of the isolates in this cluster were recovered in a single month (June 2017). These findings suggest that temporal collection gaps did not substantially bias the overall phylogenetic structure of the reduced dataset.

### 3.6. B. cereus Isolates from Toronto Show Genetic Diversity Comparable to a Global Collection of Isolates

Taken together, the previous findings, including the open pangenome revealed by rarefaction analysis, the high diversity observed in accessory gene content, and the lack of clustering by isolate source in earlier phylogenetic analyses, highlight the relatively high level of genetic diversity within *B. cereus* isolates collected in Toronto, Canada. To better understand how this diversity compares globally, we included a representative collection of *B. cereus* sensu lato genomes (n = 373) downloaded from NCBI. These genomes span over 70 years [28] and originate from eight countries: Argentina, Brazil, Canada, China, Korea, Japan, the United Kingdom and the United States (Table S5).

**Figure 5:**
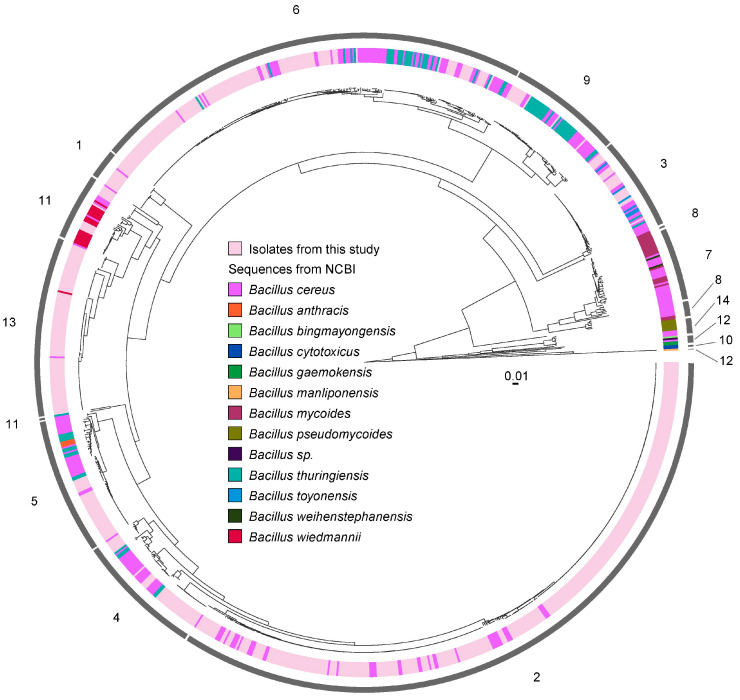
Phylogenetic relationships between *B. cereus* isolates from an HMB and a network of three downtown Toronto acute care hospitals, all served by the same microbiology laboratory, and a global *B. cereus* collection. The genetic relationships among 688 *B. cereus* isolates from this study are illustrated, alongside 373 *B. cereus* sensu lato genomes obtained from NCBI (Table S5), collected over 70 years and spanning eight countries (Argentina, Brazil, Canada, China, Korea, Japan, the United Kingdom and the United States). The phylogenetic tree was constructed using a recombination-removed and gap-free core genome alignment (136,810 nt). Statistical support for tree splits was assessed with the Shimodaira–Hasegawa test (1000 resamples) in FastTree 2.1. Hierarchical clustering analysis identified 14 clusters, including one heterogeneous cluster (cluster 11), as shown in the outermost ring. Colors in the second outermost ring represent the origin (local or global) and taxonomic assignment of genomes.

Phylogenetic analysis using a recombination-removed and gap-free core genome alignment revealed that *B. cereus* isolates from the premises of the Toronto HMB and 3 downtown Toronto hospitals were interspersed throughout the global dataset (Figure 5), with the exception of clusters 7, 8, 10, 12, and 14, which contained no isolates from Toronto. However, these clusters were mainly formed by non-*B. cereus* sensu stricto isolates (Figure 5). The interspersion of local and global *B. cereus* isolates noted on the remaining nine clusters (Figure 5) recapitulates the lack of distinct clustering by source or geographic origin, indicating that the *B. cereus* genetic diversity that is observed locally is comparable to that found globally across strains spanning several decades and multiple countries.

The largest BAPS cluster was cluster 6, comprising 232 isolates, of which 137 originated from our collection (Figure 5). This cluster included isolates from all three source categories: human milk (n = 30), patient (n = 59), and the environment (n = 48). The analysis also recapitulated the known evolutionary relationships among members of the *B. cereus* sensu lato group. Notably, genomes of *B. cereus* and *B. thuringiensis* were frequently intermixed across six clusters (3, 4, 5, 6, 9, and 11), appearing alongside both local and global *B. cereus* genomes (Figure 5). The heterogeneous nature of BAPS cluster 11 is particularly striking, as it includes genomes of *B. wiedmannii*, *Bacillus anthracis*, and *B. cereus*. Among the 12 local isolates found in this cluster, 11 were derived from patient samples. In BAPS cluster 3 (n = 23), local *B. cereus* isolates clustered alongside *Bacillus toyonensis* and *B. thuringiensis*, highlighting the overlapping core genome features across these taxa (Figure 5).

### 3.7. Identifying Genetic Determinants of Pasteurization Survival in B. cereus Isolates

**Figure 6:**
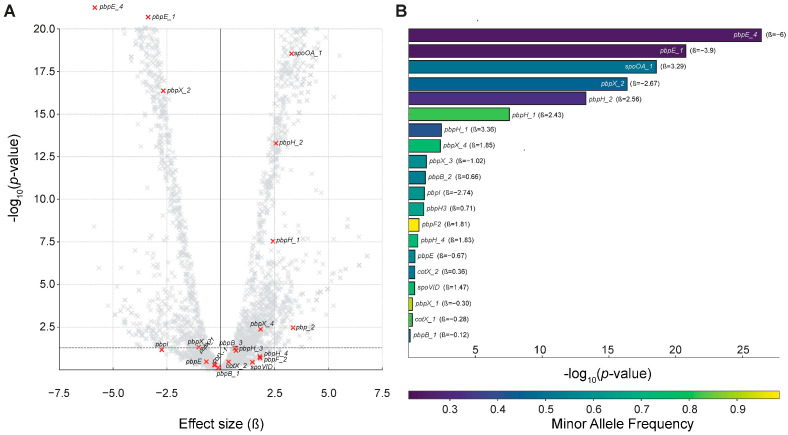
Genetic associations with *B. cereus* survival following milk pasteurization. (**A**) Volcano plot of GWAS results. Each point represents a unitig tested for association with the post-HoP phenotype among 257 *B. cereus* genomes. The x-axis indicates the estimated effect size (β), and the y-axis shows −log_10_(*p*-value). Genes related to sporulation and cell envelope function are highlighted in red and labeled. Axes were capped at β = ±7.5 and −log_10_(*p*) = 20 to improve readability. Dashed lines represent the nominal significance threshold (*p* = 0.05) and zero effect. (**B**) Focused view of the loci involved in sporulation and cell envelope biology. Barplot of selected genes previously implicated in endospore formation (e.g., *spoVID*, *spo0A*, and *cotX*) or involved in peptidoglycan biosynthesis (e.g., penicillin-binding protein-encoding genes or *pbps*) that were associated with post-HoP survival. Bars are ordered by −log_10_(*p*-value) and colored according to minor allele frequency (MAF); effect size estimates (β) are provided alongside gene names. Lower MAF values indicate that the associated variant is rarer across the population, possibly reflecting recent acquisition, strain specificity, or subpopulation adaptation.

Previous phylogenetic analyses demonstrated substantial genetic diversity across *B. cereus* isolates from the Toronto HMB, but did not reveal clear phylogenetic distinctions between isolates from different sources. Specifically, post-HoP milk isolates were interspersed among pre-HoP and other environmental or patient-derived isolates. Post-HoP milk isolates represent a subset of the *B. cereus* population that has survived a rigorous selective pressure. Thus, to investigate potential genetic determinants of HoP resilience not captured in phylogenetic analysis, we performed GWAS. Using the reduced collection of 257 genomes, including 98 post-HoP isolates, GWAS was conducted to identify unitigs significantly associated with the post-pasteurization phenotype. The heritability of this phenotype was estimated at 0.99, suggesting that up to 99% of its variation could be explained by genetic differences captured in the dataset. The full association landscape is shown in the volcano plot in Figure 6A. Significant unitigs were annotated through mapping to a reference genome and, where applicable, through additional functional annotation databases and genome assemblies, since no single genome captures the full diversity of the *B. cereus* pangenome. While many of the most statistically significant loci encoded hypothetical proteins, several genes involved in sporulation and cell envelope function also showed strong associations and are highlighted in the plot.

To focus the analysis on these biologically meaningful candidates, we selected a subset of genes for visualization in Figure 6B. This included known contributors to sporulation (e.g., *spoVID*, *spo0A*, *cotX*) and cell wall integrity (e.g., *pbpE*_*1*, *pbpH*_*2*). Although these loci were not always among the top 25 ranked hits by p-value, they were significantly associated with the post-pasteurization phenotype and represent plausible contributors to heat resilience. The use of a unitig-based GWAS approach enabled the detection of intra-gene sequence variation, which is often masked by clustering algorithms in pangenomic analyses such as Roary, which collapse sequences sharing >90% identity. Thus, variants present within widely distributed genes, rather than gene presence/absence per se, likely drive survival-associated phenotypes.

Several of the highlighted variants displayed low to moderate minor allele frequencies (MAFs), suggesting they may represent adaptations restricted to a subset of isolates. This is consistent with a model in which post-pasteurization survival reflects the localized or recent genetic changes within generally conserved gene frameworks. For example, *spoVID* and *pbp* genes were present across most isolates, but specific unitigs representing distinct variants were disproportionately enriched in genomes recovered from post-HoP milk. Together, these findings support the hypothesis that the selective survival of *B. cereus* following pasteurization is influenced by genetic variation affecting sporulation and envelope function. Further work will be required to test the functional effects of these variants—for example, through mutagenesis or structural modeling—and to evaluate their predictive value in screening or risk stratification tools for human milk banking protocols.

## 4. Discussion

### 4.1. Genomic Diversity and Taxonomic Complexity of B. cereus in the HMB Setting

*B. cereus* is a motile, spore-forming, Gram-positive bacterium capable of causing severe disease in vulnerable populations such as premature neonates. Its spores resist standard cleaning procedures and survive HoP, leading to the contamination of pasteurized human milk and contributing to discard rates of up to 30% at some HMBs [29,30,31,32]. Although technological alternatives such as high-pressure processing and ultraviolet-C irradiation have shown promise under experimental conditions [33,34,35], their adoption in HMBs remains limited, in part due to unresolved questions about their impact on milk quality and their cost-effectiveness [36,37]. Future studies should evaluate whether such technologies could also effectively reduce *B. cereus* contamination, particularly spore-forming strains, without compromising the nutritional and bioactive properties of donor human milk. Therefore, a better understanding of the population structure and strain diversity of *B. cereus* in the HMB setting is needed to inform more targeted and feasible risk-mitigation strategies. In this study, genomic analysis revealed that the level of *B. cereus* diversity was remarkably high, even within the restricted spatial and temporal confines of a Canadian milk bank and a geographically limited hospital network served by a single laboratory, indicating that *B. cereus* pangenome is “open” even at a single location in Canada (Figure S2). Furthermore, when we performed phylogenetic analysis using the core genome of our local isolates and a global collection of *B. cereus* sensu lato genomes available from NCBI, we found that isolates in our collection, despite being geographically and temporally restricted, contained a level of diversity comparable to that found globally over 70 years [28]. Whereas previous studies have largely relied on observational evidence from environmental sampling and conventional microbiological testing, which have a limited resolution for strain discrimination, our genomic analysis reveals the extent to which genetically diverse *B. cereus* populations can coexist even within a geographically confined hospital network and milk bank setting. While multiple potential reservoirs, such as catheters, ventilators, and linens, are frequently acknowledged in the literature [32], the broad-scale genetic heterogeneity of *B. cereus* is seldom emphasized. Our findings add an important dimension to this body of work by demonstrating that even within a confined spatial and temporal context, *B. cereus* diversity can mirror global patterns. This underscores the complexity of contamination dynamics and reinforces the need for genomic approaches in understanding and mitigating *B. cereus* contamination at human milk banks.

### 4.2. B. cereus Clonality and Plate-Level Heterogeneity

Another important contribution of this work is the evaluation of the clonality of *B. cereus* organisms derived from the same collection specimen (within plates). We found that in an all-against-all SNP distance comparison in the milk-, patient-, and environment-derived isolate categories, more than 70% of SNP distances were restricted to the range of 0–5 SNPs, and all categories followed a bimodal SNP distribution. This was evidence that, overall, *B. cereus* colonies originating from the same plate are more related to each other than to *B. cereus* colonies between plates. However, the normal microbiological practice of recovering more than one colony appears justified, since, in some plates, we recovered genetically different organisms. We have shown here that a core genome SNP cutoff of 14.5 permits differentiation between near-identical and different genomes for organisms growing from the same plate. While direct genomic studies of multiple *B. cereus* subtypes from the same plate are limited, broader research on human mastitis, as well as reports from the dairy industry, have shown that single milk- or processing-line samples often harbor genetically distinct *B. cereus*, even within the same sample [38,39]. These findings reinforce the importance of multi-colony sampling when evaluating contamination risks, particularly in milk-related settings relevant to neonatal care. Although milk dispensed by HMBs is rigorously screened, pre-pasteurization samples may harbor multiple genetically distinct *B. cereus* strains, whereas clinical infections typically involve a single strain (pure culture). While a comparison between pre- and post-pasteurization samples would have been more informative, all human milk plates were grouped for analysis due to the small number of total pre-pasteurization plates in the dataset. Multiple colonies were sampled from 39 of 98 milk plates (39.8%), revealing substantial genetic diversity, with within-plate pairwise SNP distances ranging from 0 to over 72,000 (Table S4). This underscores the necessity of sampling more than one colony per plate when attempting to trace potential sources of infection. Notably, wide within-plate SNP variation was observed among milk-derived isolates (mean = 4105 SNPs; SD = 9530), confirming that selecting a single colony would have missed substantial strain diversity in many cases.

### 4.3. Multifactorial Sources of B. cereus Contamination and Practical Mitigation

Despite the lack of organization and structure at isolation source level, hierarchical Bayesian analysis of a phylogenetic tree constructed from core genome SNPs revealed a highly ordered underlying *B. cereus* population structure composed of nine discrete clusters. While patient-derived isolates appeared to cluster closely between themselves (e.g., within 0–12 SNPs), these typically represented multiple colonies from the same clinical isolate rather than independent infections from different patients. This tight clustering likely reflects within-isolate homogeneity rather than evidence of person-to-person transmission or the selection of particularly virulent genotypes. We also identified strong clustering of post-HoP milk-derived isolates, which suggests that the selection pressures introduced by HoP influences the type of *B. cereus* organisms that are recovered post-pasteurization; however, the clusters of post-HoP milk-derived isolates belonged to different phylogenetic clades. Although our findings underscore the widespread environmental presence of *B. cereus*, we also sought to determine whether there was genomic evidence pointing towards the survival of spores through pasteurization or recontamination post-HoP. To explore this hypothesis, we examined SNP-based clustering and phylogenetic relationships among isolates. In several cases, post-HoP isolates clustered closely (≤15 SNPs) with pre-HoP or environmental isolates, consistent with the survival of clonal strains through the HoP process, possibly as spores. In contrast, other post-HoP isolates showed no close relationship with any pre-HoP strain, suggesting the possibility of recontamination during or after milk-handling. These complementary patterns support the likely coexistence of both mechanisms, i.e., spore-mediated persistence and post-HoP contamination, within our dataset. A key limitation of our study, however, is the absence of matched pre- and post-HoP isolates from the same milk pool or donor batch. Although milk was sampled before and after pasteurization, the lack of linked identifiers across sampling points prevented direct strain-level comparisons that could have more definitively distinguished between these two scenarios.

Consistent with these findings, patient-derived isolates and environmental isolates were interspersed in these same clusters, making it difficult to pinpoint the source of contamination. The absence of source-specific clustering underscores the multifactorial nature of contamination and emphasizes the importance of the rigorous standards already in place for donor milk-processing. Current practices, including hygienic expression protocols, equipment sterilization, and donor-screening, form an essential foundation for minimizing the initial bacterial load. Maintaining these high standards, alongside environmental monitoring and regular workflow reviews, may further enhance the ability to detect and mitigate rare or unpredictable sources of contamination, particularly in processing and storage areas [36,37]. Moreover, the implementation of predictive microbiological risk models may allow for real-time adjustments in response to contamination trends [29]. Furthermore, our findings show that *B. cereus* is not only a significant environmental contaminant in HMB but also in the NICUs. Although there is no evidence linking these settings to neonatal patient infections, continued environmental monitoring and regular workflow reviews may reinforce the strong safeguards already in place to protect these vulnerable patients.

### 4.4. Potential Genetic Determinants for B. cereus Survival in Post-Pasteurization Milk

While our phylogenetic analyses revealed that *B. cereus* isolates from pre- and post-HoP were interspersed with environmental and patient-derived strains, the persistence of certain lineages in HoP-treated milk suggested that specific genetic factors may confer a survival advantage. One plausible source of such factors is extrachromosomal DNA: *B. cereus* can harbor large plasmids exceeding 600 kbp, but current short-read sequencing typically fails to circularize plasmids over 5 kbp, making it difficult to determine whether key survival genes reside on chromosomal or plasmid DNA [40]. To circumvent these limitations, we performed a GWAS based on unitigs (high-resolution sequence fragments capable of capturing allelic variation within widely distributed genes). This analysis, summarized in Figure 6, revealed significant associations between the post-pasteurization phenotype and loci involved in sporulation and cell envelope biology. Among them were penicillin-binding protein variants, spore coat protein X (*cotX*), and *spoVID*-dependent spore coat assembly factors. While not always the top-ranked hits according to *p*-value, these genes were consistently enriched in post-HoP isolates and selected for focused presentation based on functional relevance. Although sporulation in *B. cereus* remains incompletely understood, these unitig-level associations suggest that certain alleles may enhance spore coat formation or stability at 62.5 °C, promoting survival during HoP. This hypothesis aligns with prior work on *Bacillus subtilis*, in which mutations in *spoVID*, *cotX*, and penicillin-binding proteins were linked to altered spore morphology and thermal resistance [41,42,43].

## 5. Conclusions

In this study, we characterized the population of *B. cereus* contaminating human milk at a major Canadian HMB, revealing an unexpectedly high level of genetic diversity, comparable to that observed in global collections. This diversity, coupled with the lack of source-specific clustering in phylogenetic analyses, highlights the multifactorial nature of *B. cereus* contamination. Our data also suggest that *B. cereus* isolates surviving HoP may harbor gene variants linked to sporulation and cell wall integrity. In particular, unitig-level associations pointed to specific sequence variants in genes such as penicillin-binding proteins, spore coat protein X, and SpoVID-dependent spore coat assembly factors, which may contribute to enhanced resistance to heat treatment. These findings highlight both the complexity of the contamination pathways and the possible biological basis for post-pasteurization survival.

Moving forward, future work integrating long-read sequencing, functional assays of spore coat formation, and experimental pasteurization models will be essential to clarify the roles of these genetic variants. A better understanding of *B. cereus* heat-resistance mechanisms could inform targeted mitigation strategies in HMBs, which currently discard up to 30% of donated milk following post-HoP *B. cereus* detection [29,30,31,37]. Identifying and monitoring heat-resistant variants more precisely may help optimize pasteurization protocols and reduce human milk loss. More broadly, combining genomic insights with robust monitoring systems and adaptive processing strategies will be key to ensuring both the safety and availability of donor milk for vulnerable infants.

## Data Availability

All sequencing data generated in this study have been deposited in the NCBI Sequence Read Archive under BioProject accession number PRJNA666097. A complete list of individual genome accession numbers is provided in Supplementary Table S2.

## Supplementary Materials

The following supporting information can be downloaded at: https://www.mdpi.com/article/10.3390/microorganisms13051136/s1. Figure S1: Flowchart of *B. cereus* collection quality checks and filters to form the reduced sets; Figure S2: Pan-genome accumulation curve and power-law regression fit for 688 *B. cereus* isolates used in this study; Table S1: Temporal and source breakdown of *Bacillus cereus* isolates analyzed in this study; Table S2: Line list of *Bacillus cereus* isolates used in this study; Table S3: Gene distribution across presence thresholds in the *Bacillus cereus* pangenome; Table S4: Summary of *Bacillus cereus* isolate genetic diversity by specimen type; Table S5: NCBI RefSeq accession numbers for genomes (n = 373) used in global diversity analysis.

## Abbreviations

The following abbreviations are used in this manuscript:

AUC	Area under the curve
BAPS	Bayesian analysis of population structure
CDS	Coding DNA Sequence
DNA	Deoxyribonucleic acid
GWAS	Genome-wide association study
HGT	Horizontal gene transfer
HMB	Human milk bank
HoP	Holder pasteurization
IQR	Interquartile range
MAF	Minor allele frequency
MALDI-TOF MS	Matrix-assisted laser desorption/ionization-time of flight mass spectrometry
MGE	Mobile genetic elements
MSH	Mount Sinai Hospital
NCBI	National Center for Biotechnology Information
NICU	Neonatal intensive care unit
ROC	Receiver operating characteristic
SD	Standard deviation
SNP	Single-nucleotide polymorphism
WGS	Whole-genome sequencing
