# Supplementary material for: High Genetic Diversity Among Bacillus cereus Isolates Contaminating Donated Milk at a Canadian Human Milk Bank
Source: Microorganisms. 2025 May 15;13(5):1136. doi: 10.3390/microorganisms13051136 (PMC12114557; doi:10.3390/microorganisms13051136)
Supplement: Supplementary file 1 [file microorganisms-13-01136-s001.zip › Figure_S1.pdf]

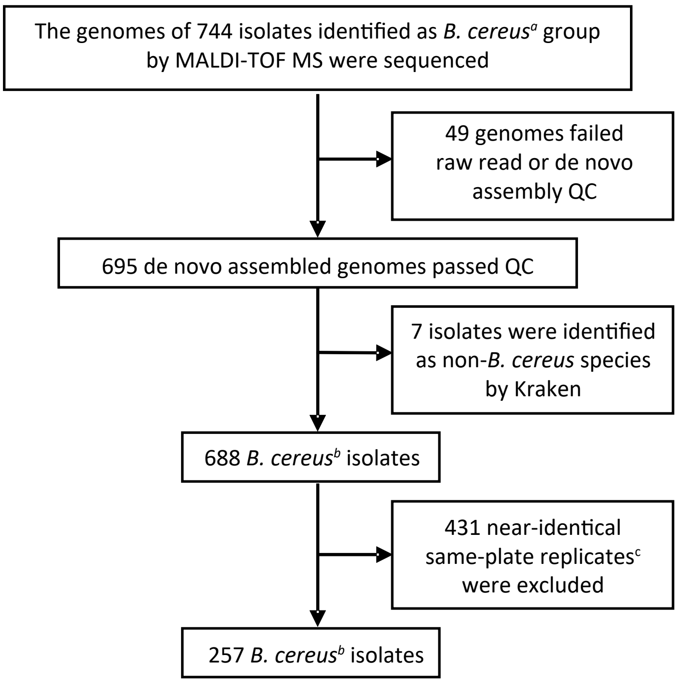

[a]Identified as *B. cereus sensu lato* isolates via MALDI-TOF MS

[b]Identified as *B. cereus sensu stricto*, *B. wiedmanii*, or *B. thuringiensis* via Kraken (taxonomically labels short-reads)

[c]Isolates from the same plate whose pairwise SNP distances were ≤ 14.5 were considered near-identical

Figure S1. Flowchart of *B. cereus* collection quality checks and filters to form the reduced sets.
