# Supplementary material for: High Genetic Diversity Among Bacillus cereus Isolates Contaminating Donated Milk at a Canadian Human Milk Bank
Source: Microorganisms. 2025 May 15;13(5):1136. doi: 10.3390/microorganisms13051136 (PMC12114557; doi:10.3390/microorganisms13051136)
Supplement: Supplementary file 1 [file microorganisms-13-01136-s001.zip › Figure_S2.pdf]

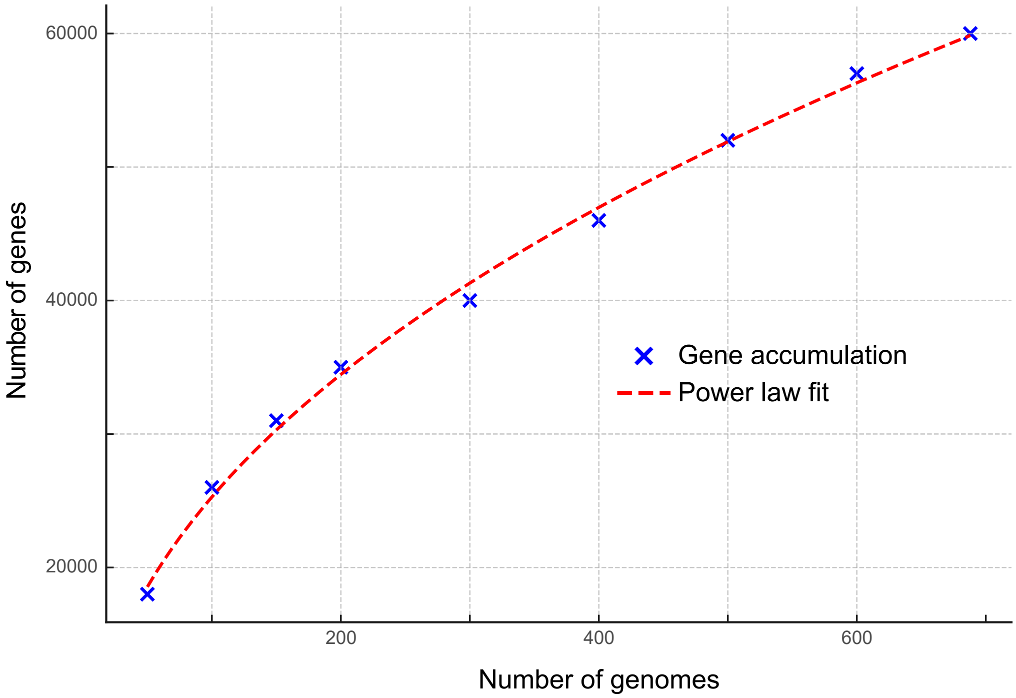

Figure S2. Pan-genome accumulation curve and power-law regression fit for 688 *B. cereus* isolates used in this study. For visual clarity, only a representative subset of gene accumulation values is shown as blue points. A power-law model of the form $Ps=\kappa n^{\gamma}$ was fitted to the full gene accumulation trend and is displayed as a dashed red line, where Ps is the total number of genes (pangenome size), n is the number of genomes analyzed, κ is a fitted constant, and γ is the exponent that determines the openness of the pan-genome. Values of $\gamma < 1$ indicate an open pan-genome in which new genes continue to be discovered as additional genomes are included, while $\gamma > 1$ would suggest a closed pangenome. The fitted γ value (~0.447), estimated from our gene accumulation data, supports the conclusion that the *B. cereus* pangenome is open.
