# Supplementary material for: High Genetic Diversity Among Bacillus cereus Isolates Contaminating Donated Milk at a Canadian Human Milk Bank
Source: Microorganisms. 2025 May 15;13(5):1136. doi: 10.3390/microorganisms13051136 (PMC12114557; doi:10.3390/microorganisms13051136)
Supplement: Supplementary file 1 [file microorganisms-13-01136-s001.zip › Table_S1.pdf]

Table S1. Temporal and source breakdown of *Bacillus cereus* isolates analyzed in this study.

Date of collection	No. of isolates	Milk (*n*=213)		Patient (*n*=349)		Environmental (*n*=126)				
		Pre-HoP[a]	Post-HoP	Colonization	Clinical	HMB[b]	Breast Pads	NICU[c]	MicroLab[d]	Other Wards
**2017-Feb**	1		1							
**2017-Mar**	1						1			
**2017-May**	4	4								
**2017-Jun**	6	1	5							
**2017-Jul**	127		10	4	12	20		71	10	
**2017-Aug**	275	1	42	182	49				1	
**2017-Sep**	79	4	11	31	16		15		1	1
**2017-Oct**	30	3	6		20				1	
**2017-Nov**	11	3			8					
**2017-Dec**	15	1	12	1	1					
**2018-Jan**	49	2	44		3					
**2018-Feb**	5	1	3		1					
**2018-Mar**	30	1	17		11					1
**2018-Apr**	18	1	9		8					
**2018-May**	37		31		2					4
**Total**	**688**	**22**	**191**	**218**	**131**	**20**	**16**	**71**	**13**	**6**

[a] HoP: Holder pasteurization.
[b] HMB: Roger Hixon Human Milk Bank premises.
[c] NICU: Mount Sinai Hospital neonatal intensive care unit.
[d] MicroLab: Mount Sinai Hospital Microbiology Laboratory.
