# Supplementary material for: High Genetic Diversity Among Bacillus cereus Isolates Contaminating Donated Milk at a Canadian Human Milk Bank
Source: Microorganisms. 2025 May 15;13(5):1136. doi: 10.3390/microorganisms13051136 (PMC12114557; doi:10.3390/microorganisms13051136)
Supplement: Supplementary file 1 [file microorganisms-13-01136-s001.zip › Table_S3.pdf]

Table S3. Gene distribution across presence thresholds in the *Bacillus cereus* pangenome

Gene category	Present in	No. of genes
Core genes	99% <= strains <= 100%	1,933
Accessory genes		61,266
Shared accessory	≥ 2 strains; part of accessory	49,727
Unique accessory	Only 1 strain	14,479
Soft core genes	95% <= strains < 99%	328
Shell genes	15% <= strains < 95%	6,797
Cloud genes	0% <= strains < 15%	54,141
Total genes	0% <= strains <= 100%	63,199
