# Supplementary material for: High Genetic Diversity Among Bacillus cereus Isolates Contaminating Donated Milk at a Canadian Human Milk Bank
Source: Microorganisms. 2025 May 15;13(5):1136. doi: 10.3390/microorganisms13051136 (PMC12114557; doi:10.3390/microorganisms13051136)
Supplement: Supplementary file 1 [file microorganisms-13-01136-s001.zip › Table_S4.pdf]

Table S4. Summary of *Bacillus cereus* isolate genetic diversity by specimen type.

Characteristics	Milk (*n*=213)	Patient (*n*=349)	Environmental (*n*=126)	Overall
Total no. of plates	98	105	23	226
No. of multi-isolate plates	39	67	17	123
No. of single-isolate plates	59	38	6	103
No. of isolates on multi-isolate plates (min – max)	2 – 5	2 – 5	3 – 15	2 – 15
Within-plate pairwise SNP distances				
Mean (± SD)	4,105 (9,530)	1.3 (1.4)[a]	10,841 (14,381)	2,823 (8,353)
Min – max	0 – 67,564	0 – 61,346	0 – 72,733	0 – 72,733
Between-plate pairwise SNP distances				
Mean (± SD)	23,027 (22,727)	39,202 (26,940)	47,252 (24,861)	36,691 (24,981)
Min – max	0 – 84,289	0 – 93,630	0 – 96,134	0 – 96,134
Within-plate pairwise accessory similarity				
Mean (± SD)	0.805 (0.179)	0.921 (0.096)	0.760 (0.188)	0.862 (0.155)
Min – max	0.155 – 0.973	0.453 – 0.981	0.158 – 0.975	0.155 – 0.981
Between-plate pairwise accessory similarity				
Mean (± SD)	0.373 (0.279)	0.452 (0.262)	0.284 (0.181)	0.329 (0.211)
Min – max	0.083 – 0.973	0.111 – 0.974	0.114 – 0.953	0.0826 – 0.974

[a] One outlier isolate was removed (SNP distance = 61,346).
