# Supplementary material for: High Genetic Diversity Among Bacillus cereus Isolates Contaminating Donated Milk at a Canadian Human Milk Bank
Source: Microorganisms. 2025 May 15;13(5):1136. doi: 10.3390/microorganisms13051136 (PMC12114557; doi:10.3390/microorganisms13051136)
Supplement: Supplementary file 1 [file microorganisms-13-01136-s001.zip › Table_S5.pdf]

Table S5. NCBI RefSeq accession numbers for genomes (*n*=373) used in global diversity analysis adapted from Bazinet, 2017. Cluster no. denotes the results from rhierBAPS.

RefSeq assembly accession	Cluster no.
GCA_000160995	1
GCA_000161075	2
GCA_000161195	3
GCA_000167215	4
GCA_000171035	2
GCA_000181595	5
GCA_000181635	5
GCA_000181655	5
GCA_000468975	6
GCA_000500585	4
GCA_000732525	7
GCA_000948355	8
GCA_001402735	9
GCA_001584085	9
GCF_000003645	2
GCF_000003925	7
GCF_000003955	8
GCF_000007825	6
GCF_000007845	5
GCF_000008005	4
GCF_000008165	5
GCF_000008445	5
GCF_000008505	5
GCF_000011625	5
GCF_000013065	2
GCF_000015065	5
GCF_000017425	10
GCF_000018825	7
GCF_000021205	6
GCF_000021225	2
GCF_000021305	9
GCF_000021785	5
GCF_000022505	5
GCF_000092165	6
GCF_000143605	5
GCF_000160895	6
GCF_000160915	5
GCF_000160935	6
GCF_000160955	11
GCF_000160975	7
GCF_000161015	4
GCF_000161035	6
GCF_000161055	6

RefSeq assembly accession	Cluster no.
GCF_000161095	7
GCF_000161115	6
GCF_000161135	5
GCF_000161155	3
GCF_000161175	6
GCF_000161215	3
GCF_000161235	5
GCF_000161255	12
GCF_000161275	6
GCF_000161295	3
GCF_000161315	6
GCF_000161335	7
GCF_000161355	6
GCF_000161375	13
GCF_000161395	8
GCF_000161415	14
GCF_000161435	14
GCF_000161455	14
GCF_000161475	4
GCF_000161495	9
GCF_000161515	9
GCF_000161555	6
GCF_000161575	6
GCF_000161595	5
GCF_000161615	9
GCF_000161635	5
GCF_000161655	5
GCF_000161675	6
GCF_000161695	5
GCF_000161715	9
GCF_000161735	9
GCF_000181615	6
GCF_000190515	4
GCF_000193355	9
GCF_000239195	5
GCF_000256545	2
GCF_000283675	2
GCF_000290655	7
GCF_000290675	7
GCF_000290695	7
GCF_000290715	6
GCF_000290735	6
GCF_000290755	6
GCF_000290775	6
GCF_000290795	6

RefSeq assembly accession	Cluster no.
GCF_000290815	3
GCF_000290835	3
GCF_000290855	8
GCF_000290875	2
GCF_000290895	7
GCF_000290915	7
GCF_000290935	2
GCF_000290955	9
GCF_000290975	7
GCF_000290995	2
GCF_000291015	3
GCF_000291035	9
GCF_000291055	8
GCF_000291075	7
GCF_000291095	3
GCF_000291115	3
GCF_000291135	5
GCF_000291155	7
GCF_000291175	7
GCF_000291195	9
GCF_000291215	6
GCF_000291235	2
GCF_000291255	6
GCF_000291275	2
GCF_000291295	7
GCF_000291315	7
GCF_000291335	7
GCF_000291355	7
GCF_000291375	11
GCF_000291395	3
GCF_000291415	6
GCF_000291435	6
GCF_000291455	6
GCF_000291475	8
GCF_000291495	11
GCF_000291515	8
GCF_000291535	6
GCF_000291665	8
GCF_000292415	4
GCF_000292455	9
GCF_000292705	9
GCF_000293505	3
GCF_000293525	3
GCF_000293545	7
GCF_000293565	11

RefSeq assembly accession	Cluster no.
GCF_000293605	7
GCF_000293685	6
GCF_000293705	7
GCF_000293725	9
GCF_000293745	3
GCF_000299035	12
GCF_000300475	3
GCF_000306745	9
GCF_000338315	4
GCF_000338755	6
GCF_000341665	9
GCF_000342025	9
GCF_000387405	9
GCF_000398785	9
GCF_000398945	9
GCF_000398965	7
GCF_000398985	7
GCF_000399005	9
GCF_000399025	6
GCF_000399045	7
GCF_000399065	9
GCF_000399085	9
GCF_000399105	9
GCF_000399125	6
GCF_000399145	7
GCF_000399165	8
GCF_000399185	9
GCF_000399205	2
GCF_000399225	2
GCF_000399245	7
GCF_000399265	9
GCF_000399285	11
GCF_000399305	9
GCF_000399325	6
GCF_000399345	6
GCF_000399365	6
GCF_000399385	3
GCF_000399405	3
GCF_000399425	7
GCF_000399445	3
GCF_000399465	8
GCF_000399485	14
GCF_000399505	6
GCF_000399525	7
GCF_000399545	6

RefSeq assembly accession	Cluster no.
GCF_000399565	6
GCF_000399585	14
GCF_000399605	14
GCF_000399625	11
GCF_000399645	11
GCF_000412315	3
GCF_000412975	9
GCF_000440035	2
GCF_000440055	2
GCF_000468995	6
GCF_000482345	12
GCF_000496285	3
GCF_000497525	9
GCF_000503755	9
GCF_000513155	7
GCF_000517985	7
GCF_000518025	6
GCF_000530375	6
GCF_000571955	9
GCF_000585975	9
GCF_000600315	6
GCF_000635895	6
GCF_000688755	4
GCF_000688795	6
GCF_000710255	6
GCF_000712595	12
GCF_000712615	12
GCF_000717445	#N/A
GCF_000742855	14
GCF_000743195	2
GCF_000743815	6
GCF_000743865	2
GCF_000746865	4
GCF_000746925	8
GCF_000746965	14
GCF_000747545	6
GCF_000773635	6
GCF_000775975	7
GCF_000789315	4
GCF_000803665	6
GCF_000816555	6
GCF_000832385	5
GCF_000832405	5
GCF_000832485	5
GCF_000832525	4

RefSeq assembly accession	Cluster no.
GCF_000832605	7
GCF_000832765	5
GCF_000832805	4
GCF_000832825	5
GCF_000832845	4
GCF_000832865	5
GCF_000832885	14
GCF_000832925	5
GCF_000833045	5
GCF_000833085	5
GCF_000833655	9
GCF_000835025	9
GCF_000835185	5
GCF_000835235	6
GCF_000878525	6
GCF_000931375	7
GCF_000935235	6
GCF_000940785	9
GCF_000948235	9
GCF_000948245	3
GCF_000948325	4
GCF_000948345	6
GCF_000969665	6
GCF_000978375	6
GCF_000987785	4
GCF_001008565	11
GCF_001008575	2
GCF_001008585	2
GCF_001008595	6
GCF_001008645	5
GCF_001008655	11
GCF_001008665	6
GCF_001008695	2
GCF_001017635	6
GCF_001043655	6
GCF_001044475	2
GCF_001044485	6
GCF_001044555	4
GCF_001044565	11
GCF_001044575	11
GCF_001044585	5
GCF_001044635	2
GCF_001044645	6
GCF_001044655	6
GCF_001044665	6

RefSeq assembly accession	Cluster no.
GCF_001044715	6
GCF_001044735	2
GCF_001044745	11
GCF_001044775	3
GCF_001044795	2
GCF_001044815	11
GCF_001044825	2
GCF_001044855	6
GCF_001044875	9
GCF_001044895	2
GCF_001044905	4
GCF_001044935	7
GCF_001044955	6
GCF_001050335	10
GCF_001058585	6
GCF_001059175	6
GCF_001059825	4
GCF_001059875	4
GCF_001077095	6
GCF_001182785	9
GCF_001183785	6
GCF_001238465	3
GCF_001275045	3
GCF_001276195	6
GCF_001277915	9
GCF_001296435	9
GCF_001317525	11
GCF_001420855	6
GCF_001455345	11
GCF_001484805	7
GCF_001518875	6
GCF_001541985	7
GCF_001541995	5
GCF_001548175	6
GCF_001566355	4
GCF_001566365	2
GCF_001566375	2
GCF_001566385	2
GCF_001566435	2
GCF_001566445	2
GCF_001566455	2
GCF_001566465	2
GCF_001566515	2
GCF_001566525	2
GCF_001566535	2

RefSeq assembly accession	Cluster no.
GCF_001566705	6
GCF_001573165	4
GCF_001579695	6
GCF_001583685	9
GCF_001583695	11
GCF_001583705	2
GCF_001583745	6
GCF_001583755	3
GCF_001583765	4
GCF_001583805	5
GCF_001583845	6
GCF_001583855	5
GCF_001583865	6
GCF_001583875	6
GCF_001583925	4
GCF_001583935	6
GCF_001583955	7
GCF_001583975	4
GCF_001584005	4
GCF_001584025	3
GCF_001584035	13
GCF_001584065	7
GCF_001584095	4
GCF_001584105	5
GCF_001590835	12
GCF_001595725	6
GCF_001598095	9
GCF_001604665	4
GCF_001618665	6
GCF_001619285	6
GCF_001619335	7
GCF_001619355	13
GCF_001619385	2
GCF_001619395	6
GCF_001619405	6
GCF_001619425	11
GCF_001619445	6
GCF_001619465	2
GCF_001619505	7
GCF_001619525	6
GCF_001635915	9
GCF_001635955	9
GCF_001635995	6
GCF_001640965	9
GCF_001645395	11

RefSeq assembly accession	Cluster no.
GCF_001645405	11
GCF_001645445	11
GCF_001645455	11
GCF_001645465	11
GCF_001645505	11
GCF_001645525	11
GCF_001645535	11
GCF_001645555	11
GCF_001677055	6
GCF_001677965	6
GCF_001678165	9
GCF_001683695	4
GCF_001683705	6
GCF_001685565	9
GCF_001692675	6
